# Changes in Urinary Hydrogen Peroxide and 8-Hydroxy-2′-Deoxyguanosine Levels after a Forest Walk: A Pilot Study

**DOI:** 10.3390/ijerph15091871

**Published:** 2018-08-29

**Authors:** Da-Hong Wang, Ai Yamada, Masamitsu Miyanaga

**Affiliations:** Department of Biochemistry, Okayama University of Science, Okayama, Okayama Prefecture 700-0005, Japan; s18bm03ya@ous.jp (A.Y.); miyanaga@dbc.ous.ac.jp (M.M.)

**Keywords:** forest walk, urban walk, H_2_O_2_, 8-OHdG, oxidative biomarker

## Abstract

Some studies have shown that exposure to forests has positive effects on human health, although the mechanisms underlying the health benefits of a forest environment have not been elucidated yet. The current study was aimed at examining how the levels of urinary hydrogen peroxide (H_2_O_2_) and 8-hydroxy-2’deoxyguanosine (8-OHdG) change after a forest or urban walk in healthy subjects. Twenty-eight volunteers (19 men and 9 women) participated in the study. The forest walks were carried out in a forest in Okayama Prefecture, Japan, and the urban walks (15 men and 7 women) were carried out in the downtown area of Okayama city, each for two hours. Spot urine samples were collected before the walk, the next day and one week after the forest or urban walk. Compared with pre-forest walk levels, urinary H_2_O_2_ (*p* < 0.1) and 8-OHdG (*p* < 0.1) concentrations significantly decreased in the participants the day after the forest walk; furthermore, urinary 8-OHdG remained at a low level even at one week after the forest walk (*p* < 0.05). However, there were no significant changes in the concentrations of these oxidative biomarkers after the urban walk. These findings suggest the possibility that exposure to forests may alleviate oxidative stress in the body.

## 1. Introduction

In recent years, several researchers have reported that people who live in areas with more green space have fewer cardiometabolic diseases and a lower mortality risk, a decreased risk of poor mental health and cardiovascular diseases and a decreased risk of hyperlipidemia [1,2,3], although the mechanisms underlying the health benefits of a forest environment have not been elucidated yet. Some reports from Japan demonstrated that a forest walk had positive effects on human physiological functions, such as a decrease in blood pressure among hypertensive patients [4,5,6] and an increase in human natural killer (NK) cell activity and anticancer protein expression [7]. An early study found a decreased lipid peroxide level in the urine immediately after a forest walk [8]. A recent report by Im et al. showed that 2 h exposure to a forest environment increased the serum levels of glutathione peroxidase, an important antioxidant enzyme in the body [9]. It is known that reactive oxygen species (ROS) are produced as byproducts of normal metabolic processes in all aerobic organisms. When ROS generation exceeds the capacity of antioxidant defense systems in the body to remove them, this imbalance can cause oxidative damage to cellular constituents (DNA, proteins, lipids, etc.), which is defined as oxidative stress [10,11]. Measuring oxidative stress biomarkers is considered to be useful for health risk prediction and disease prevention [12,13]. However, few studies have examined the oxidative and antioxidative biomarkers in the body after exposure to a forest environment [8,9,14]. Moreover, there was no report on how the levels of urinary hydrogen peroxide (H_2_O_2_) change after a forest walk, and there was disagreement regarding the levels of urinary 8-hydroxy-2′deoxyguanosine (8-OHdG) after a forest walk. Accordingly, the current information, which includes a small sample size and a few oxidative biomarkers, is insufficient to substantiate the effect of the forest environment on regulating the oxidant/antioxidant imbalance in humans and more evidence is highly desirable. It is well-known that the fresh green odor emitted by trees and plants contains organic volatile compounds called phytoncides [15]. Phytoncide extracts from trees have shown antioxidant effects [16]; some studies found that phytoncide attenuated lipopolysaccharide-induced inflammatory responses by decreasing oxidative stress generation in macrophage cells (RAW264.7) and bovine mammary epithelial cells [17,18]. Accordingly, we hypothesized that exposure to the forest environment might regulate the oxidant/antioxidant imbalance in humans. In the current study, we used urine-based biomarkers for a noninvasive evaluation of how the levels of oxidative biomarkers would be altered after a forest or urban walk.

## 2. Subjects and Methods

### 2.1. Study Environments

**Figure 1:**
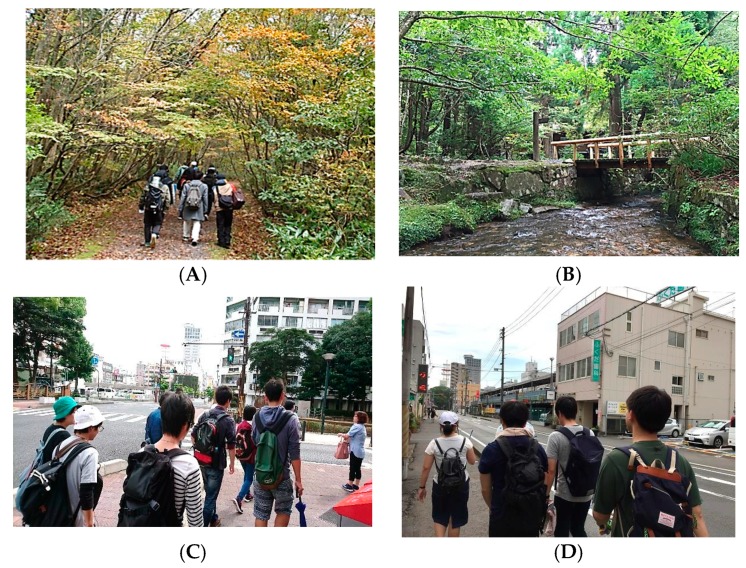
(**A**,**B**) Pictures of the forest walk. (**C**,**D**) Pictures of the urban walk.

The forest walks were carried out in Okayama Prefecture Forest Park (total area: 3.34 km^2^), located in the northern Okayama Prefecture, Japan (Figure 1A,B). The park abounds with broad-leaved trees, such as Japanese beech (*Fagus crenata*), Mizunara (*Quercus crispula*), and maple (*Acer*) trees. It took about 2 h to drive from Okayama city to the park. All the participants were accompanied by laboratory staff; they went to the park in a chartered bus and then took a walk together for 2 h. The urban walks were carried out in the downtown area of Okayama (Figure 1C,D), which has a population of 720,066 and a total area of 789.95 km^2^. For the urban walks, all the participants gathered in front of the Okayama railway station and took a walk together around the downtown area for 2 h. There were two–three short breaks during both the forest and the urban walks. The forest walks were carried out in 2015–2017 and the urban walks were added to the study in 2016–2017 (Table 1).

During the walks, air temperature, relative humidity and atmospheric pressure at five sites were measured by a humidity/barometer/temperature data recorder (MHB-382SD, Sato Shouji Inc., Tokyo, Japan), wind speed was measured using an anemometer (MODEL6006-31, Kanomax, Osaka, Japan) and illuminance was measured by a light-emitting diode light meter (TM-209 M, FUSU Rika Corporation, Tokyo, Japan).

### 2.2. Participants and Sample Preparation

A total of 28 senior university students voluntarily participated in the forest walks, including 9 students in 2015, nine students in 2016, and 10 students in 2017; and 22 students participated in the urban walks, including 10 students in 2016 and 12 students in 2017 (Table 1). The studies conducted in 2016 and 2017 had a crossover design, and all the participants in each year attended both the forest and urban walks in 1 group, except that 1 person was absent from the forest walk in 2016 because of an unexpected poor body condition and 2 persons were absent from the forest walk in 2017 because of an important job interview (Table 2). The participants had the same lunch on the days of the forest and urban walks.

Two to three days before the walk, the next day, and 1 week after the forest or urban walk, a blood pressure measurement, spot urine collection and questionnaire survey were carried out. Blood pressure was measured in a sitting position after resting for at least 10 min, using an automatic blood pressure monitor (OMRON, Kyoto, Japan). We chose 3 sampling days (before, the next day and 1 week later) because NK cell activity and anticancer proteins maintained higher levels on day 1 and day 7 after forest bathing [19,20]. Spot urine samples (midstream urine) were collected and were stored at –80 °C until analysis. The samples were centrifuged at 5000 rpm for 5 min at 4 °C to remove cellular fractions. Supernatants were used for the analysis. All the participants were asked not to drink coffee, tea or alcohol and not to smoke before the urine sampling. Information on lifestyle, including cigarette smoking, alcohol consumption, exercise/physical activity and vegetable/fruit consumption was obtained using a self-reported questionnaire.

The study protocol was approved by the Ethics Committee of the Okayama University of Science (No. 27-4). Written informed consent was obtained from all the participants.

### 2.3. Analysis of Urinary Oxidative Biomarkers

Urinary hydrogen peroxide (H_2_O_2_) was measured using the method of ferrous ion oxidation in the presence of xylenol orange, version 1 (FOX-1) [21,22]. In brief, urine samples (20 μL) were incubated with the same volume of either a catalase solution (2200 U/mL in 25 mM phosphate buffer, pH 7.0) or 25 mM phosphate buffer (pH 7.0). The samples were then reacted with 160 μL of the FOX-1 reagent (100 μM xylenol orange, 100 mM sorbitol, 250 μM ammonium ferrous sulfate, and 25 mM H_2_PO_4_; pH adjusted to 1.7–1.8 through the addition of Na_2_HPO_4_) at room temperature for 30 min. The absorbance was measured with a microplate reader (SH-1200; Corona Electric Co., Ltd., Tokyo, Japan) at 560 nm. The concentration of H_2_O_2_ was calculated from the difference in absorbance (with and without catalase) using a standard curve. Urinary H_2_O_2_ was expressed as micromoles per gram of creatinine.

Urinary 8-hydroxy-2′-deoxyguanosine (8-OHdG) was analyzed by a specific enzyme-linked immunosorbent assay kit according to the manufacturer’s protocol (Japan Institute for the Control of Aging, Shizuoka, Japan) [23]. Urinary 8-OHdG was expressed as nanograms per milligram of creatinine.

Urinary creatinine was measured by a commercial alkaline picrate reagent colorimetric assay at 490 nm (R&D Systems, Minneapolis, MN, USA) and the values of the urinary biomarkers were normalized to those of urinary creatinine.

### 2.4. Statistical Analysis

All the data are expressed as the mean ± standard error of the mean (SEM). Log-transformed urinary H_2_O_2_ and 8-OHdG data were used for all the statistical analyses because of their skewed distributions. The urinary H_2_O_2_ and 8-OHdG levels were compared using a paired *t*-test (pre-walk vs. the day after the walk; pre-walk vs. one week after the walk), and the results were considered to be significant at the 10% level [24]. The data analysis was performed using the IBM SPSS Statistics Package version 22 for Windows (SPSS Inc., Chicago, IL, USA).

## 3. Results

### 3.1. Environmental Measures

Table 1 shows the results of environmental measures, including air temperature, relative humidity, wind speed, atmospheric pressure and illuminance.

### 3.2. Characteristics of Participants

The profiles of the participants are shown in Table 2. All the participants were senior university students in their twenties. More than 60% were men in both the forest and urban walk groups. About 14 or 18% of the participants were current smokers; less than 10% drank alcohol regularly; less than 10 or 20% engaged in exercise/physical activity; about 30 or 40% consumed vegetables regularly, and about 10% consumed fruit regularly.

### 3.3. Decreased Levels of Urinary H_2_O_2_ and 8-OHdG after a Forest Walk

**Figure 2:**
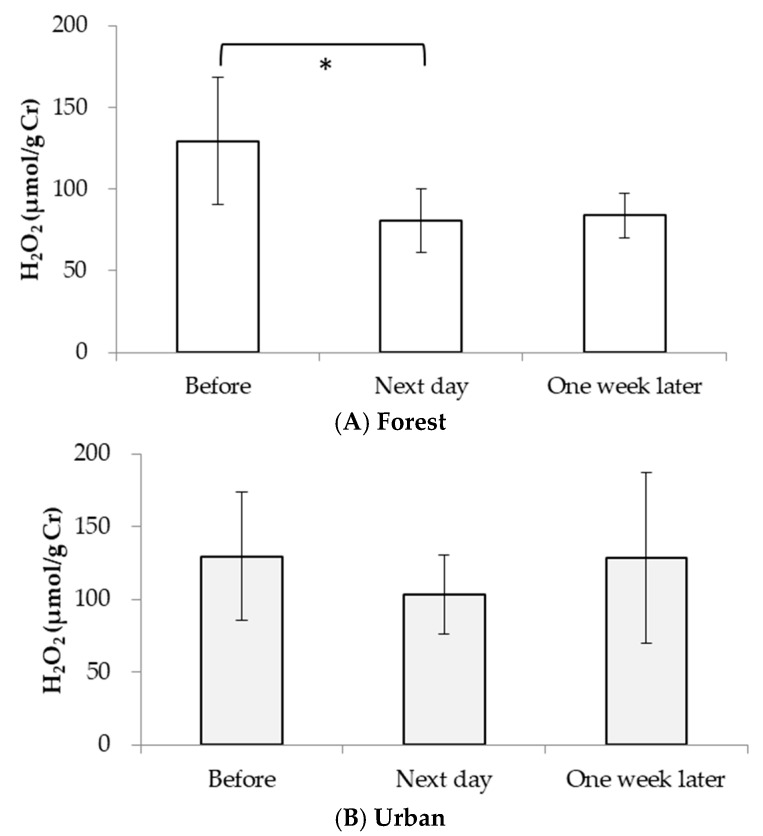
(**A**) Urinary H_2_O_2_ levels before, the next day and one week after the forest or urban walk (* *p* < 0.1: pre-forest walk vs. the day after the forest walk). (**B**) Urinary H_2_O_2_ levels before, the next day and one week after the urban walk.

Figure 2A shows that compared with the concentration of pre-forest walking, the mean concentration of urinary H_2_O_2_ significantly decreased the day after the forest walk (*p* < 0.1). However, there was no significant change in urinary H_2_O_2_ levels after the urban walk (Figure 2B).

**Figure 3:**
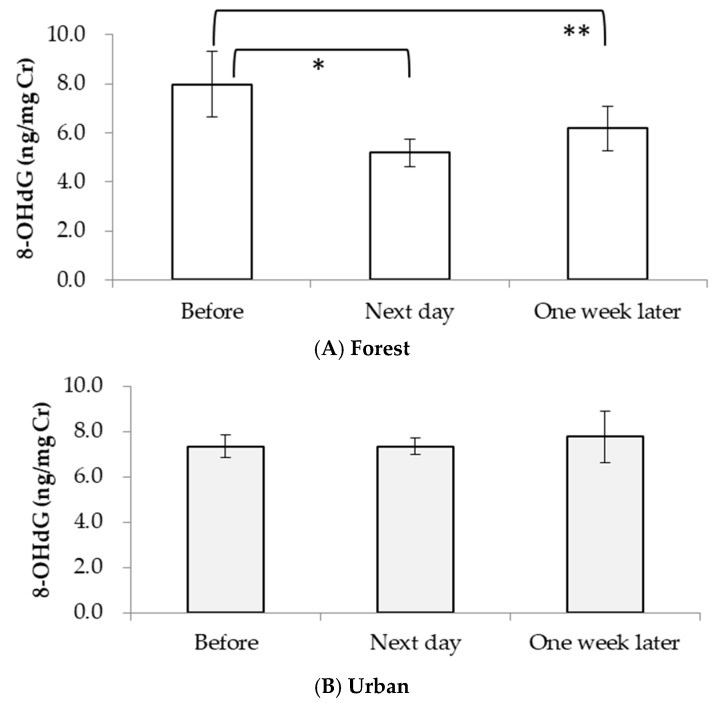
(**A**) Urinary 8-OHdG levels before, the next day and one week after the forest or urban walk (* *p* < 0.1: pre-forest walk vs. the day after the forest walk; ** *p* < 0.05: pre-forest walk vs. one week after the forest walk). (**B**) Urinary 8-OHdG levels before, the next day and one week after the urban walk.

The mean concentration of 8-OHdG also significantly decreased the next day (*p* < 0.1) and was still low one week after (*p* < 0.05) the forest walk compared with the value pre-walk (Figure 3A). We did not observe any significant changes in urinary 8-OHdG levels after the urban walk (Figure 3B).

## 4. Discussion

This is the first report of decreased urinary H_2_O_2_ levels after a forest walk. Hydrogen peroxide is a byproduct of oxidative metabolism [25,26,27,28] and is also known as an ROS. Although H_2_O_2_ itself is chemically less reactive, it is able to diffuse across the cell membrane and form other highly reactive intermediates, such as the hydroxyl radical (OH・), in the presence of trace iron or copper to induce cell damage [28]. Im et al. reported that 2 h exposure to a forest environment increased serum glutathione peroxidase (GPx) levels [9]. GPx is an endogenous antioxidant enzyme that degenerates H_2_O_2_ in the biological system; the lower H_2_O_2_ levels after forest walks observed in our study might be partially explained by the enhanced antioxidative capacity in the body. The current study also observed a 23.2% decrease in urinary potential antioxidant levels (PAO) one week after the 2 h urban walk compared with the level pre-walk (*p* < 0.1 by paired t-test), although we did not find a significant increase in the PAO level the day after the forest walk (Supplementary Figure S1). Some published works have demonstrated that green plants release various phytoncides [15], such as α-pinene, limonene and careen, which showed a radical elimination action [16] by decreasing superoxide peroxidation and increasing Nrf2 activation in mammary alveolar epithelial cells [18]. Moreover, phytoncide exposure not only elevated NK activity and the percentage of NK cells [29] but also reduced ROS-induced cytotoxicity [16,30]. The forest environment contains a great deal of phytoncides released by green plants. The concentrations of α-pinene, β-pinene and limonene in the forest are ten times higher than in the urban environment [31]. In the urban environment, traffic-related air pollutants like sulfate and O_3_ are associated with oxidative stress in humans [32]. Therefore, exposure to a forest environment may play some role in alleviating the generation of oxidative stress in biological systems.

It is well-known that 8-OHdG is produced by the oxidative modification of the DNA base guanine through the OH・attack generated by H_2_O_2_ in a biological system [28]. The decreased H_2_O_2_ level (Figure 2A) after a forest walk is expected to result in a decrease in OH・generation. Our results show that the 8-OHdG levels not only significantly decreased on the day after the forest walk but were still low one week later compared with the pre-forest walk levels, which differs from previous studies [8,14]. The work by Hayashi et al. (*n* = 9) showed an increased urinary 8-OHdG level after a 30-min walk in both forest and urban environments [14], and the study by Gibo et al. (*n* = 10) found no significant change in urinary 8-OHdG after a daily 3–4-km walk in the forest for two days [8]. Such disagreements might be partially due to differences such as walking duration and sample size. Further studies with an increased sample size and varied walk duration are needed to answer this question.

The current study contributes to natural greenery literature regarding the health benefits of forest walks. However, this pilot study has limitations, such as the small sample size and young participants. Therefore, caution should be taken to avoid generalization of the findings. In addition, the level of statistical significance of our results was 10%; at this level, only small study effects might be detected. To confirm the current results, further studies with an increased sample size are warranted. In this study, spot urine was sampled instead of a 24-h urine collection, because Møller and Loft reported a strong correlation (*r* = 0.87) in 8-OHdG levels measured by enzyme-linked immunosorbent assay between spot and 24-h urine samples [33]. We did not check for women’s menstrual cycles; however, Asare et al. reported that the levels of urinary oxidative markers were not affected by the ovulatory and luteal phases of the menstrual cycle among healthy women [34]. Further studies are needed to confirm the current findings and to evaluate the alteration of other oxidative or antioxidative biomarkers after a forest walk.

## 5. Conclusions

This pilot study provides evidence that compared with pre-forest walk levels, urinary H_2_O_2_ and 8-OHdG concentrations significantly decreased in the participants after the forest walk. This was not the case for urban walks. The current findings, however preliminary, suggest that forest walking may play a role in the alleviation of oxidative stress in the body.

## Figures and Tables

**Table 1:** Characteristics of the environment.

Parameter	Forest Environment			Urban Environment	
	October 2015	September 2016	October 2017	July 2016	September 2017
Air temperature (°C)	17.7~21.3	22.6~23.3	10.8~13.3	33.3~36.3	25.5~28.8
Relative humidity (%)	39.3~60.7	81.3~88.9	65.2~74.7	49.5~59.8	61.8~70.3
Wind speed (m/s)	0.25~1.19	0.30~1.05	0.10~1.75	1.20~2.67	0.33~1.62
Atmospheric pressure (hPa)	916.7~923.1	912.8~919.5	918.2~924.0	888.7~1011.4	1007.1~1008.6
Illuminance (lx)	702~97,200	1746~12,130	1325~25,430	20,200~52,800	421~3875
Elevation (m)	840~900	840~900	840~960	2	2

Five sites were measured during each walk. The minimum and maximum values of the data are given.

**Table 2:** Participants’ demographics.

Parameter	No. (%) or Mean ± SEM	
	Urban (*n* = 22)	Forest (*n* = 28)
Sex		
Male	–	7 (14 October 2015)
	6 (4 July 2016)	6 (28 September 2016)
	9 (27 September 2017)	7 (25 October 2017) ^a^
Female	–	2 (14 October 2015)
	4 (4 July 2016)	3 (28 September 2016) ^b^
	3 (27 September 2017)	3 (25 October 2017)
BMI (kg/m^2^)	22.3 ± 0.8	22.8 ± 0.8
Smoker		
No	18 (81.8)	22 (78.6)
Current	3 (13.6)	5 (17.9)
Past	1 (4.6)	1 (3.5)
Alcohol consumption		
None	10 (45.5)	14 (50.0)
<3 times/week	10 (45.5)	12 (42.9)
≥4 times/week	2 (9.0)	2 (7.1)
Exercise/physical activity		
No	20 (90.9)	23 (82.1)
Yes	2 (9.1)	5 (17.9)
Vegetable consumption		
Almost none	1 (4.5)	2 (7.1)
Sometimes	15 (68.2)	15 (53.6)
A little per day	6 (27.3)	11 (39.3)
A lot per day	0	0
Fruit consumption		
Almost none	7 (31.8)	8 (28.6)
Sometimes	13 (59.1)	17 (60.7)
A little per day	2 (9.1)	3 (10.7)
A lot per day	0	0
SBP (mmHg)	116 ± 1	121 ± 1
DBP (mmHg)	68 ± 1	72 ± 1

BMI, SBP and DBP data are expressed as mean ± standard error of the mean (SEM). BMI, body mass index; SBP, systolic blood pressure; DBP, diastolic blood pressure. ^a^ Two subjects could not attend the forest walk because of job interviews. ^b^ One subject could not attend the forest walk because of an unexpected poor body condition.

## Supplementary Materials

The following are available online at http://www.mdpi.com/1660-4601/15/9/1871/s1, Figure S1: Urinary potential antioxidant level (PAO) before, the next day and one week after forest or urban walks (* *p* < 0.1 by a paired *t*-test: pre-urban walk vs. one week after the urban walk).
