# Supplementary material for: Changes in Urinary Hydrogen Peroxide and 8-Hydroxy-2′-Deoxyguanosine Levels after a Forest Walk: A Pilot Study
Source: Int J Environ Res Public Health. 2018 Aug 29;15(9):1871. doi: 10.3390/ijerph15091871 (PMC6163805; doi:10.3390/ijerph15091871)
Supplement: Supplementary file 1 [file ijerph-15-01871-s001.pdf]

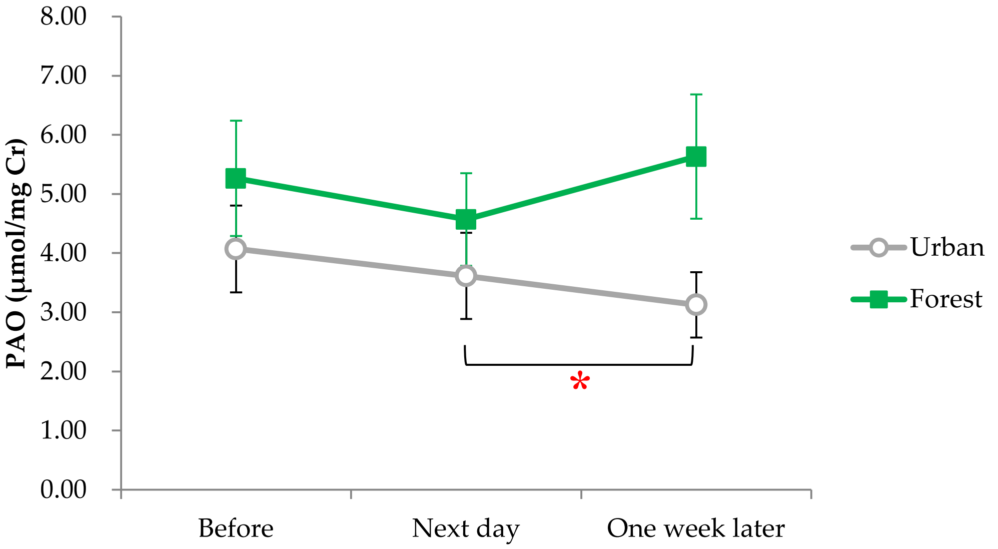

Figure S1: Urinary potential antioxidant level (PAO) before, the next day, and 1 week after forest or urban walks (*$p < 0.1$ by a paired t-test: pre–urban walk vs. 1 week after the urban walk).
